# High level of drug resistance by gram-negative bacteria from selected sewage polluted urban rivers in Addis Ababa, Ethiopia

**DOI:** 10.1186/s13104-018-3622-0

**Published:** 2018-07-31

**Authors:** Teshome Belachew, Amete Mihret, Tesfaye Legesse, Yihenew Million, Kassu Desta

**Affiliations:** 1 Department of Medical Microbiology, School of Laboratory Medicine, College of Medicine and Health Sciences, University of Gondar, 196, Gondar, Ethiopia; 2 Ethiopian Public Health Institute, Addis Ababa, Ethiopia; 3 Department of Medical Laboratory Sciences, College of Health Sciences, Addis Ababa University, Addis Ababa, Ethiopia

**Keywords:** River water, Gram-negative, Drug resistance, Addis Ababa

## Abstract

**Objective:**

The aim of this study was to determine the level of drug resistance by gram-negative bacteria isolated from selected sewage polluted urban rivers in Addis Ababa, Ethiopia.

**Results:**

From a total of 94 river water samples, 90 medically important gram-negative bacterial isolates were recovered to the species level. The predominant bacteria isolated were *E. coli*. 23 (26%) followed by *K. pneumoniae* 18 (20%), *K. oxytoca* 17 (19%). *E. coli* showed a high level of resistance to ampicillin 21 (91.3%), cefalotin, cefuroxime, ceftriaxone and cefepime 16 (70%). Both *K. pneumoniae* and *K. oxytoca* showed high resistance to ampicillin 16 (94%) and 17 (95%) respectively. Among identified bacterial species, most of them showed a multidrug-resistant pattern. Providential retigerri showed 100% multidrug resistance followed by *P. alkalificiens* (90%), *E. coli* (78%), *M. morgani* (75%), and *C. frundi* (60%).

**Electronic supplementary material:**

The online version of this article (10.1186/s13104-018-3622-0) contains supplementary material, which is available to authorized users.

## Introduction

Unprotected water sources can be contaminated with microbes through different factors. But, sewage effluents and healthcare institutions are the most possible causes of contamination of rivers, wells, and dams. This makes them unacceptable for human consumption because of harboring both pathogenic and commensal bacteria [1].

The majority of antibiotics used in human and animal healthcare is partially metabolized and released through excreta into the municipal sewage system. Untreated liquid waste containing partially metabolized antibiotics in low concentration contributes largely to the development of antibiotic resistance in the environmental microflora [2–5]. If hospitals and municipal sewage effluents are not treated, infectious agents and antibiotic-resistant microbes are shed into rivers and finally reach into communities. As the result, people will acquire waterborne diseases such as cholera, typhoid fever, dysentery and gastroenteritis, which cannot be treated by conventional antibiotics [6].

Nowadays, the spread of antimicrobial resistance (AMR) among pathogenic and commensal bacteria is a global health concern. WHO estimates that about 1.1 billion people globally, drink unsafe water and the vast majority of diarrheal disease in the world (88%) is attributable to unsafe water sanitation and hygiene. Each year, about 17 million people die of infectious diseases worldwide, most of which are caused by bacteria [7]. CDC has also stated that in the United States, more than two million people are sickened and 23,000 die each year as a result of antibiotic-resistant infections [8]. Antibiotic resistance can lead to increases in human and animal health care costs as well as increased mortality and morbidity [9]. To the best of our knowledge, there is scarce information with regard to the level of bacterial profiles and antimicrobial resistance from rivers of Addis Ababa, Ethiopia.

## Main text

### Materials and methods

#### Study area and setting

The study was conducted on selected polluted urban rivers that pass through ten sub-cities of Addis Ababa, Ethiopia.

#### Study design and period

A cross sectional study design was employed from February to April, 2017.

#### Sampling methods and procedures

Rivers that have large water streams were selected from each sub-cities of Addis Ababa. About 32 rivers were included for this study. Grab sampling technique was employed for sample collection. From 10 rivers, three discrete water samples (1 from first sampling point, 1 from 100 m downstream and 1 from 200 m downstream) were collected from each river in the first round and processed independently. In the second round, two discrete water samples (1 from first sampling point and 1 from 100 m downstream) were collected and processed independently. From the remaining 22 rivers, a single discrete water sample was collected from each river in the first and second round. Second round samples were collected with 15 days interval. A total of 94 river water samples was collected. All samples were manually collected by using actual sample containers and then transported immediately to the Ethiopian Public Health Institute (EPHI) Microbiology Laboratory with cold chain. A clean gown and pair of new, non-powdered, disposable gloves were used each time while collecting water samples from different rivers often sub-cities. All samples were processed within 4 h of collection. About 150 ml sterile glass containers were used to collect 100 ml water samples.

#### Sample processing, isolation, and identification of bacteria

The water samples were diluted tenfold serially with sterile physiological saline. Then, 5–20 µl of the aliquot was used to streak blood and MacConkey (Oxoid, UK) agar then incubated aerobically at 37 °C for 24–48 h [10]. On the following day, based on colony morphology and other important features, colonies from primary media were sub-cultured [11–13]. Presumptively isolated organisms were further identified to species level by BioMerieux VITEK 2 Compact system using the ID-GN cards. Once a species identification was done, confirmed species were also tested for 19 antibiotics using the Vitek 2 Compact system using the ID-GN cards. Once a species identification was done, confirmed species were also tested for 19 antibiotics using the Vitek 2 system with GN-72 cards. Results of antimicrobial susceptibility tests were interpreted based on the Clinical Laboratory Standard Institute (CLSI) guideline 2015 [14] with knowledge database of the Vitek 2 system. The multidrug-resistance (MDR) pattern was defined and interpreted based on interim standard definitions for acquiring resistance published in Clinical Microbiology and Infection and formulated by a group of international experts [15].

### Results

#### Pattern of bacterial isolates

A total of 150 bacterial isolates were recovered to the species level. Of these, 60 bacterial isolates were not medically important and 90 bacterial isolates were medically important. So, non medically important bacterial isolates were left out of this paper and mainly focused on medically important bacterial isolates. The bacterial isolation rate was 98% (i.e. only two water samples showed no growth, but all 32 rivers were positive for one or more than one bacterial species). Among medically important 90 bacterial isolates, *E. coli* was the predominant which accounted 23 (26%) followed by *K. pneumoniae* 18 (20%), and *K. oxytoca* (19%). The least identified bacterial species were *M. morganii* 4 (4%) (Table 1).

**Table 1:** Frequency of gram negative bacterial isolates from Urban rivers in Addis Ababa, Ethiopia, June, 2017

Bacterial isolates	Frequency	Percent (%)
*E. coli*	23	26
*K. pneumoniae*	18	20
*K. oxytoca*	17	19
*P. alcalifaciens*	10	11
*C. Freundii*	10	11
*P. rettgeri*	8	9
*M. morganii*	4	4
Total	90	100%

#### Antimicrobial susceptibility patterns of bacterial isolates

The predominant isolate, *E. coli*, showed a high level of resistance to many of tested antibiotics like ampicillin 21 (91%), cefalotin, cefuroxime, ceftriaxone, cefepime each accounted 16 (70%). Both *K. pneumoniae* and *K. oxytoca* showed high resistance to ampicillin, with the resistance rate of 17 (95%) and 16 (94%) respectively. Another bacterial isolate, *C. frundi* showed very high resistance to cefazolin, amoxicillin–clavulanic acid and cefuroxime; 8 (80%), 7 (70%), 7 (70%) respectively. However, all isolates were 0% resistant to ceftriaxone, tetracycline, nitrofurantoin and trimethoprim–sulfamethoxazole (Table 2 and Additional file 1: Table S1).

**Table 2:** Antimicrobial susceptibility patterns of gram-negative bacterial isolates from river water, June, 2017, Addis Ababa Ethiopia

Antimic.	*E. coli* (No. = 23)			*K. pnumoniae* (No. = 18)			*K. oxytoca* (No. = 17)			*C. freundii* (No. = 10)		
	S	I	R	S	**I**	R	S	**I**	R	S	**I**	R
AM	2 (9)	0 (0)	21 (91)	1 (6)	0 (0)	17 (94)	0 (0)	1 (6)	16 (94)	0 (0)	1 (10)	3 (30)
AMC	13 (57)	1 (4)	12 (52)	14 (78)	2 (11)	2 (11)	12 (71)	2 (12)	3 (18)	3 (30)	0 (0)	7 (70)
TZP	11 (48)	1 (4)	10 (43)	16 (89)	0 (0)	2 (11)	13 (76)	1 (6)	3 (18)	4 (40)	0 (0)	4 (40)
CF	6 (26)	1 (4)	16 (70)	14 (78)	1 (6)	3 (17)	9 (53)	2 (12)	6 (35)	2 (20)	0 (0)	6 (60)
CZ	8 (35)	1 (4)	14 (61)	15 (83)	0 (0)	3 (17)	12 (71)	0 (0)	5 (29)	1 (10)	1 (10)	8 (80)
CXM	6 (26)	0 (0)	16 (70)	13 (72)	0 (0)	5 (28)	11 (65)	0 (0)	6 (35)	2 (20)	0 (0)	7 (70)
CXMAX	6 (26)	3 (13)	14 (61)	12 (67)	0 (0)	6 (33)	11 (65)	1 (6)	5 (29)	3 (30)	1 (10)	5 (50)
FOX	10 (43)	1 (4)	10 (43)	14 (78)	1 (6)	3 (17)	14 (82)	0 (0)	3 (18)	4 (40)	1 (10)	4 (40)
CPD	5 (22)	0 (0)	17 (74)	13 (72)	0 (0)	3 (17)	12 (71)	0 (0)	5 (29)	5 (50)	0 (0)	3 (30)
CAZ	7 (30)	1 (4)	15 (65)	13 (72)	1 (6)	3 (17)	11 (65)	0 (0)	6 (35)	8 (80)	0 (0)	2 (20)
CRO	5 (22)	0 (0)	16 (70)	13 (72)	0 (0)	4 (22)	11 (65)	0 (0)	6 (35)	6 (60)	0 (0)	4 (40)
FEP	6 (26)	1 (4)	16 (70)	13 (72)	0 (0)	5 (28)	11 (65)	0 (0)	6 (35)	6 (60)	0 (0)	4 (40)
GM	11 (48)	2 (9)	10 (43)	15 (83)	0 (0)	3 (17)	13 (76)	0 (0)	4 (24)	7 (70)	0 (0)	3 (30)
TM	14 (61)	0 (0)	9 (39)	13 (72)	1 (6)	4 (22)	13 (76)	0 (0)	4 (24)	7 (70)	0 (0)	2 (20)
CIP	10 (43)	1 (4)	12 (52)	14 (78)	1 (6)	3 (17)	13 (76)	2 (12)	2 (12)	6 (60)	1 (10)	3 (30)
LEV	11 (48)	2 (9)	8 (35)	15 (83)	0 (0)	3 (17)	15 (88)	0 (0)	2 (12)	8 (80)	0 (0)	1 (10)
TE	9 (39)	0 (0)	14 (61)	11 (61)	1 (6)	6 (33)	13 (76)	1 (6)	3 (18)	6 (60)	0 (0)	4 (40)
FT	17 (74)	1 (4)	4 (17)	9 (50)	5 (28)	3 (17)	14 (82)	2 (12)	1 (6)	6 (60)	3 (30)	1 (10)
SXT	10 (43)	0 (0)	13 (67)	13 (72)	0 (0)	5 (28)	11 (65)	0 (0)	6 (35)	6 (60)	0 (0)	4 (40)

*AM* ampicillin, *AMC* amoxicillin/clavulnic acid, *TZP* piperacillin/tazobactam, *CF* cefalothin, *CZ* cefazolin, *CXM* cefuroxime, *CXMAX* cefuroxime axetil, *FOX* cefoxitin, *CPD* cefpodoxime, *CAZ* ceftazidime, *CRO* ceftriaxone, *FEP* cefepime, *GM* gentamicin, *TM* tobramycin, *CIP* ciprofloxacin, *LEV* levofloxacin, *TE* tetracycline, *FT* nitrofurantoin, *SXT* trimethoprim/sulfamethoxazole

#### Multidrug resistance patterns of the bacterial isolates

Among identified bacterial species, most of them were multidrug resistant (MDR). *P. rettgeri* showed 100% multidrug resistance followed by *P. alcalifaciens* (90%), *E. coli* (78%), *M. morganii* (75%) and *C. frundii* (60%). Only 5% of the total isolates were 0% resistant to all tested antibiotics (Table 3).

**Table 3:** Multidrug resistance patterns of gram negative bacteria isolated from selected rivers in Addis Ababa, Ethiopia June, 2017

Bacterial isolate	Number of antimicrobials resisted no (%)									
	R0	R1	R2	R3	R4	R5	R6	R7	Total	MDR
*E. coli* (N = 23)	1 (4)	1 (4)	3 (13)	2 (9)	3 (13)	6 (26)	6 (26)	1 (4)	23 (100)	18 (78)
*K. pneumoniae* (N = 18*)*	1 (6)	7 (39)	3 (17)	2 (11)	1 (6)	3 (17)	1 (6)	0 (0)	18 (100)	7 (39)
*K. oxytoca* (N = 17)	0 (0)	7 (41)	3 (18)	3 (18)	2 (12)	1 (6)	0 (0)	1 (6)	17 (100)	7 (41)
*C. freundi* (N = 10)	0 (0)	2 (20)	2 (20)	1 (10)	4 (40)	1 (10)	0 (0)	0 (0)	10 (100)	6 (60)
*P. alcalifaciens* (N = 10)	0 (0)	1 (10)	0 (0)	2 (20)	2 (20)	5 (50)	0 (0)	0 (0)	10 (100)	9 (90)
*P. rettgeri* (N = 8)	0 (0)	0 (0)	0 (0)	1 (13)	4 (50)	2 (25)	0 (0)	1 (13)	8 (100)	8 (100)
*M. morganii* (N = 4)	0 (0)	1 (25)	0 (0)	2 (50)	1 (25)	0 (0)	0 (0)	0 (0)	4 (100)	3 (75)
Total	2 (2)	19 (21)	11 (12)	12 (13)	15 (17)	18 (20)	7 (8)	3 (3)	90 (100)	58 (64)

*R0* no resistance for any class of antimicrobial, *R1* resistance for one class of antimicrobials, *R2* resistance for two class of antimicrobial, *R3* resistance for three class of antimicrobials, *R4* resistance for four class of antimicrobials, *R5* resistance for five class of antimicrobials, *R6* resistance for six class of antimicrobials, *R7* resistance for seven class of antimicrobials

### Discussion

Bacteriological analyses of river water are used to assess its quality for human consumption, recreational purpose or agricultural activities to safeguard public health. The majority of the river water sources harbored enteric pathogens and were also reported to be of poor microbiological quality and unsafe for consumption [16]. The presence of enteric bacterial pathogens in water sources may spell health hazards such as diarrheal diseases, which accounts for a substantial degree of morbidity and mortality in adults and children [17]. Management of diarrhea may require the administration of antibiotics. However, several bacteria are known to be resistant to a wide array of antibiotics [18]. The high rate of bacterial isolation from rivers precludes from direct domestic use and may also be problematic for flocculation and filtration purposes, with a consequent increase in the cost of water treatment. To the best of our knowledge, this is the first study in Ethiopia, which attempted to assess microbiological quality of river water, and antibiotic susceptibility of gram-negative bacterial isolates.

In this study, assessing the gram-negative bacterial profile and antibiotic susceptibility patterns of river water sources were examined in order to establish the physical safety of water sources and to provide updated data on antibiograms of enteric and non-enteric pathogens for better management of patients requiring empiric antibiotic therapy.

The isolation rate of gram-negative bacteria from this study was 98%, which is almost in agreement with other studies done in Spain, Romania, South Africa and Nigeria in which, all (100%) of the samples were positive for one or more than one bacterial isolates [18–21]. Among medically important 90 isolated gram-negative bacterial species, *E. coli* was the predominant bacteria accounted 23 (26%) followed by *K. pneumoniae* 18 (20%), *K. oxytoca* 17 (19%) *C. freundii* 10 (11%) and *P. alcalifaciens* 10 (11%) which is, in accordance of other studies done in Romania, South Africa and Mexico [19, 20, 22].

*Escherichia coli* showed a high level of resistance to many of these tested antibiotics like ampicillin 21 (91%), cefalotin, cefuroxime, ceftriaxone, and cefepime each accounted 16 (70%). This was higher than previously reported results in Mexico (39.4, 36.4, 12.1, 12.1 and 12.1% respectively) [22]. This high resistance rate for this study may result from poor waste management practice, lack of treatment plants for domestic and healthcare institutions and poor antimicrobial usage in Addis Ababa. We have noticed that none of the hospitals in Addis Ababa do have waste treatment system as a result, 78% of *E. coli* species had multi-drug resistance, which is, very high compared to previously reported results in Romania (60.34%) [19] and Netherland (11%) [23].

*Klebsiella pneumoniae* and *K. oxytoca* were another bacterial isolates abundantly identified with this study and they accounted 18 (20%) and 17 (19%) respectively. Both species showed high-level resistance to ampicillin (94%) which was higher than 63% reported in Romania [19].

Resistance to penicillin antibiotics especially resistance to ampicillin becomes very common in the world [24] and our finding is in line with this evidence.

A high rate of MDR was observed for *Klebsiella* species and the average MDR rate was 40.5% which was higher than results reported in Romania which is, 33% [19] and results reported in Spain which is (5.5%) [18]. However, MDR rate of the current finding was lower than previously reported results in Mexico (50%) [22] and results reported in Brazil (77.5%) [25]. This variation may be due to the difference in antimicrobial use and availability of waste treatment system in hospitals and other sewage.

*Citrobacter freundii* showed very high resistance to cefazolin, 8 (80%), amoxicillin/clavulanic acid 7 (70%) and cefuroxime 7 (70%). However, this result was lower than previously reported findings in Egypt; (100%) resistant to ampicillin and SXT [26].

Another gram-negative bacterial isolate, *P. rettgeri* showed high resistance to tetracycline and amoxicillin/clavulanic acid with the percentage of 7 (88%) and 6 (75%) respectively. This result was lower than reported results in Egypt (100% resistant to ampicillin and tetracycline) [26]. *P. alcalifaciens* also showed high resistance to ampicillin, tetracycline, cefazolin and nitrofurantoin with the resistance rate of 8 (80%), 8 (80%), 7 (70%) and 6 (60%) respectively. *P. rettgeri* and *P. alcalifaciens* showed MDR rate of 100 and 90% respectively.

*Morganella morganii* was another bacterial species isolated in low number with multi-drug resistance rates of 75%. We are unable to compare this finding with other literature. This is due to the fact that we have used Vitek 2 system for identification and drug resistance testing most likely includes more species compared to the conventional methods.

## Limitations of the study

Although this study addresses important public health issues, it is not free from limitation. We are unable to identify the source of the microorganisms; even though one can guess the hospital wastes could be the major sources. Parallelly, gram-positive bacteria are not included in this study. In addition, carbapenamase and extended spectrum betalactamase pattern of isolated bacterial species was not determined.

## Additional file

**Additional file 1: Table S1.** Antimicrobial Susceptibility Patterns of Gram-Negative Bacterial Isolates from river water, June, 2017, Addis Ababa Ethiopia. This table shows the antimicrobial susceptibility pattern of some gram negative bacterial species isolated from river waters. This table should be put just after Table 2 (see above the “Discussion”).

## Abbreviations

AMR: antimicrobial resistance
ATCC: American Type Culture Collection
CLSI: Clinical Laboratory Standard Institute
DRERC: Departmental Research and Ethics Review Committee
EPHI: Ethiopian Public Health Institute
MDR: multi dreg resistance
MIC: minimum inhibitory concentration
SOP: standard operating procedure
SPSS: Statistical Package for Social Science
WHO: World Health Organization
