# Supplementary material for: High level of drug resistance by gram-negative bacteria from selected sewage polluted urban rivers in Addis Ababa, Ethiopia
Source: BMC Res Notes. 2018 Jul 31;11:524. doi: 10.1186/s13104-018-3622-0 (PMC6069984; doi:10.1186/s13104-018-3622-0)
Supplement: Supplementary file 1 — Additional file 1: Table S1. Antimicrobial Susceptibility Patterns of Gram-Negative Bacterial Isolates from river water, June, 2017, Addis Ababa Ethiopia. This table shows the antimicrobial susceptibility pattern of some gram negative bacterial species isolated from river waters. This table should be put just after Table 2 (see above the “Discussion”). [file 13104_2018_3622_MOESM1_ESM.docx]

|  |  |  |  | |  | |  | Bacterial Isolates | |  | |  | | |  | | |  |
| --- | --- | --- | --- | --- | --- | --- | --- | --- | --- | --- | --- | --- | --- | --- | --- | --- | --- | --- |
|  | Antimicr | *P. alcalifaciens*(N=10) | | | | | *P. rettgeri* (N=8) | | | | | |  | *M.morganii* ( N=4) | | | | |
|  |  | S | | I | | R | S | | I | | R | |  | S | | I | R | |
|  | AM | 1(10) | | 1(10) | | 8 (80) | 2(25) | | 0 (0) | | 6 (75) | |  | 1(25) | | 0(0) | 3(75) | |
|  | AMC | 1(10) | | 0 (0) | | 2 (20) | 1 (13) | | 0 (0) | | 2 (25) | |  | 2(50) | | 0(0) | 2(50) | |
|  | TZP | 6(60) | | 1(10) | | 0 (0) | 4(50) | | 0 (0) | | 2 (25) | |  | 3(75) | | 0(0) | 1(25) | |
|  | CF | 2(20) | | 0 (0) | | 6 (60) | 3(38) | | 1 (13) | | 4 (50) | |  | 3(75) | | 0(0) | 1(25) | |
|  | CZ | 2(20) | | 0 (0) | | 7 (70) |  | | 3 (38) | | 0 (0) | |  | 2(50) | | 0(0) | 2(50) | |
|  | CXM | 4(40) | | 1 (10) | | 4 (40) | 5(63) | | 0 (0) | | 3 (38) | |  | 3(75) | | 0(0) | 1(25) | |
|  | CXMAX | 4(40) | | 0 (0) | | 5 (50) | 4(50) | | 1 (13) | | 3(38) | |  | 2(50) | | 0(0) | 2(50) | |
|  | FOX | 6(60) | | 0 (0) | | 2 (20) | 6(75) | | 0 (0) | | 1(13) | |  | 2(50) | | 0(0) | 2(50) | |
|  | CPD | 5(50) | | 2 (20) | | 3 (30) | 4(50) | | 2 (25) | | 2 (25) | |  | 2(50) | | 0(0) | 1(25) | |
|  | CAZ | 6(60) | | 1 (10) | | 3 (30) | 4(50) | | 0(0) | | 4 (50) | |  | 1(25) | | 1(25) | 2(50) | |
|  | CRO | 6(60) | | 0 (0) | | 4 (40) | 4(50) | | 2 (25) | | 1 (13) | |  | 2(50) | | 1(25) | 1(25) | |
|  | FEP | 7(70) | | 0 (0) | | 3 (30) | 7(88) | | 0 (0) | | 1(13) | |  | 3(75) | | 0(0) | 1(25) | |
|  | GM | 7(70) | | 0 (00 | | 2 (20) | 7(88) | | 0 (0) | | 1(13) | |  | 3(75) | | 0(0) | 1(25) | |
|  | TM | 9(90) | | 0 (0) | | 1(10) | 6(75) | | 0 (0) | | 2 (25) | |  | 3(75) | | 0(0) | 1(25) | |
|  | CIP | 8(80) | | 0 (0) | | 2 (20) | 5(63) | | 0 (0) | | 3 (38) | |  | 3(75) | | 0(0) | 1(25) | |
|  | LEV | 8(80) | | 0 (0) | | 2 (20) | 7(88) | | 0 (0) | | 1(13) | |  | 3(75) | | 0(0) | 1(25) | |
|  | TE | 2(20) | | 0 (0) | | 8(80) | 1(13) | | 0 (0) | | 7(88) | |  | 2(50) | | 1(25) | 1(25) | |
|  | FT | 4(40) | | 0 (0) | | 6(60) | 3(38) | | 0 (0) | | 5(63) | |  | 3(75) | | 0(0) | 1(25) | |
|  | SXT | 6(60) | | 0(0) | | 4(40) | 2(25) | | 1(0) | | 5(63) | |  | 3(75) | | 0(0) | 1(25) | |
|  |  |  | |  | |  |  | |  | |  | |  |  | |  |  | |

Table S1: Antimicrobial Susceptibility Patterns of Gram-Negative Bacterial Isolates from river water, June, 2017, Addis Ababa Ethiopia.

*AM =Ampicillin*

*AMC=Amoxicillin/Clavulnic acid TZP = Piperacillin/Tazobactam*

*CF = Cefalothin*

*CZ= Cefazolin*

*CXM = Cefuroxime*

*CXMAX= Cefuroxime Axetil*

*FOX= Cefoxitin*

*CPD = Cefpodoxime*

*CAZ= Ceftazidime*

*CRO= Ceftriaxone*

*FEP= Cefepime*

*GM= Gentamicin*

*TM =Tobramycin*

*CIP= Ciprofloxacin*

*LEV= Levofloxacin*

*TE= Tetracycline*

*FT= Nitrofurantoin*

*SXT = Trimethoprim/Sulfamethoxazole*
